# Antitumor magnetic hyperthermia induced by RGD-functionalized Fe_3_O_4_ nanoparticles, in an experimental model of colorectal liver metastases

**DOI:** 10.3762/bjnano.7.147

**Published:** 2016-10-28

**Authors:** Oihane K Arriortua, Eneko Garaio, Borja Herrero de la Parte, Maite Insausti, Luis Lezama, Fernando Plazaola, Jose Angel García, Jesús M Aizpurua, Maialen Sagartzazu, Mireia Irazola, Nestor Etxebarria, Ignacio García-Alonso, Alberto Saiz-López, José Javier Echevarria-Uraga

**Affiliations:** 1 Faculty of Science and Technology, University of the Basque Country, UPV/EHU, 48080, Bilbao, Spain; 2 Faculty of Medicine and Nursing, University of the Basque Country, UPV/EHU, P.O. Box 644, 48080, Bilbao, Spain; 3 BCMaterials, Basque Center for Materials, Applications and Nanostructures, 48160, Derio, Spain; 4 José Mari Korta Center, University of the Basque Country, UPV/EHU, 20018 Donostia, Spain,; 5 Galdakao Usansolo Hospital, 48960 Bizkaia, Spain

**Keywords:** magnetite nanoparticles, magnetic hyperthermia, RGD functionalization, tumor targeting

## Abstract

This work reports important advances in the study of magnetic nanoparticles (MNPs) related to their application in different research fields such as magnetic hyperthermia. Nanotherapy based on targeted nanoparticles could become an attractive alternative to conventional oncologic treatments as it allows a local heating in tumoral surroundings without damage to healthy tissue. RGD-peptide-conjugated MNPs have been designed to specifically target α_V_β_3_ receptor-expressing cancer cells, being bound the RGD peptides by “click chemistry” due to its selectivity and applicability. The thermal decomposition of iron metallo-organic precursors yield homogeneous Fe_3_O_4_ nanoparticles that have been properly functionalized with RGD peptides, and the preparation of magnetic fluids has been achieved. The nanoparticles were characterized by transmission electron microscopy (TEM), vibrating sample magnetometry (VSM), electron magnetic resonance (EMR) spectroscopy and magnetic hyperthermia. The nanoparticles present superparamagnetic behavior with very high magnetization values, which yield hyperthermia values above 500 W/g for magnetic fluids. These fluids have been administrated to rats, but instead of injecting MNP fluid directly into liver tumors, intravascular administration of MNPs in animals with induced colorectal tumors has been performed. Afterwards the animals were exposed to an alternating magnetic field in order to achieve hyperthermia. The evolution of an in vivo model has been described, resulting in a significant reduction in tumor viability.

## Introduction

Colorectal liver metastases are still a challenge for surgeons and oncologists. Though surgical removal of the metastases is currently the best therapeutic option, it is only indicated in less than 50% of the patients. Percutaneous ablation and arterial chemoembolization may be useful for those patients excluded from surgery [1–4]. However, in those patients with massive liver metastases only systemic chemotherapy, alone or combined with arterial radioembolization, has been reported to achieve some clinical benefits [5–6]. Hyperthermia is currently being explored as a possible new therapeutic tool for liver metastases, where it is useful as a coadjuvant for either chemotherapy or radiotherapy as it increases sensitivity for tumor cells [7–8]. Magnetic nanoparticles (MNPs) injected directly into liver tumors have become an important platform in these treatments because the application of an alternating magnetic field (AMF) increases the temperature in their near surroundings. If enough MNPs are selectively placed in the cancer tissue, it can be heated or even destroyed [9–13]. Despite the promising results obtained in these experimental settings, their clinical translation is still poor. Intravascular administration of MNPs could be an interesting approach, as it would allow a more selective and at the same time more widely spread distribution of the hyperthermic agent. As liver metastases receive their blood supply mainly from the hepatic artery, arterial administration of MNPs could achieve higher concentrations of the MNPs in the tumor than in the normal liver tissue [14]. However, it is not easy to penetrate the tumor vessels due to their irregularities and chaotic distribution. In this sense, here it is proposed that possibly disrupting the stromal surroundings of the liver metastases could seriously compromise tumor cell viability.

For such tumor destruction it is necessary to combine the heat capacity of the magnetic system with its localization in tumor tissues. However, the creation of high quality magnetic nanosystems that meet both requirements is still a great challenge. Magnetite-based systems are an interesting alternative due to the relatively high saturation magnetization, magnetic anisotropy, and lack of toxicity, together with the possibility of tailoring the dimensions, parameters which strongly influence the specific adsorption rate (SAR) [15–17]. Additionally, it is important to direct and focus these nanoparticles to the area of interest, whereby a suitable coating and functionalization of the nanoparticles is necessary [18–23]. One of the best approaches to direct nanoparticles to the target tissue is the conjugation to a tumor-specific molecule which remains on the cell surface [24–25]. The fact that RGD (arginylglycylaspartic acid) peptides are highly specific for integrins (such as α_V_β_3_ and α_V_β_5_, which are specifically expressed in tumor endothelia [26–27]) confirms the validity of this peptide as one of the most promising binding motifs. These molecules could also be easily modified with functional groups, from azides or amines to other amino acids and fluorophores that give them different specificities [28]. One of the procedures to bind RGD peptides due to its unique features such as high aqueous competency, efficiency and applicability toward diverse substrates is “click chemistry” [29–30]. Moreover, multistep reactions could be shortened by combining click chemistry with other conventional bioconjugation methods. Such strategies are commonly involved in the preparation of multifunctional nano-biohybrids with the advantage of an improvement in the selectivity of the reaction [31].

In this paper, a successive addition synthesis by a thermal decomposition method has been employed to obtain oleic acid and oleylamine-capped magnetite nanoparticles with defined sizes [32–34]. Nevertheless, these high quality, organic solvent dispersed, iron oxide nanocrystals must be transferred into aqueous phase and make them active in bioconjugation reactions with biomolecules [35]. In order to obtain the required aqueous stability, amphiphilic polymers like poly(maleic anhydride-alt-1-octadecene (PMAO) are able to form oil-in-water micelles, encapsulating iron oxide nanocrystals inside them. The monomeric units of maleic anhydride are easily hydrolyzed to form carboxylic groups, becoming hydrosoluble. Besides, the conjugation of carboxylates to a linker containing both an amino and a triple bond group generates an intermediate which could fulfill the “click criteria” to add RGD by means of 1,3-dipolar cycloaddition of azides with the terminal alkynes. The preparation, together with both structural and magnetic characterization of RGD-functionalized nanoparticles, are also presented in this paper. The ultimate goal would be to deliver enough of these RGD-functionalized MNPs through the hepatic artery to the connective tissue around the liver metastases in order to achieve a local thermal increase to destroy the vessels and hinder tumor progression. Moreover, this alteration of the tumor homeostasis could increase the efficacy of other therapeutic approaches [36].

## Results and Discussion

The method of thermal decomposition by successive additions allowed the synthesis of magnetite nanoparticles surrounded by oleic acid (Fe_3_O_4_@OA), which have been transferred to water by an amphiphilic ligand, becoming hydrosoluble nanoparticles (Fe_3_O_4_@PMAO). Carboxylic groups of PMAO were anchored with an amino-modified linker to add an alkyne group, which are the alkyne-modified NPs coupled by the azide-modified RGD peptide (RGD-N_3_) in water in a one-step procedure by a click reaction (Fe_3_O_4_@PMAO_RGD). Details of the preparation process can be found in the Experimental section below.

### Structural, morphologic and spectroscopic characterization

In order to know the characteristics and the magnetic response of the nanoparticles, X-ray diffraction (XRD), transmission electron microscopy (TEM), infrared spectroscopy (IR), dynamic light scattering (DLS) and thermogravimetric analysis (TGA) were performed.

The XRD profiles of the synthesized Fe_3_O_4_ NPs confirmed the presence of nanocrystalline structures with quite broad diffraction peaks whose positions and relative intensities match well with the standard profile of the characteristic spinel structure (JCPDS file n19-629) (Supporting Information File 1, Figure S1). From the full width at half maximum (FWHM) of the (311) diffraction peak an average crystallite size of 18 nm for the Fe_3_O_4_@OA sample was calculated by Scherrer’s formula.

**Figure 1:**
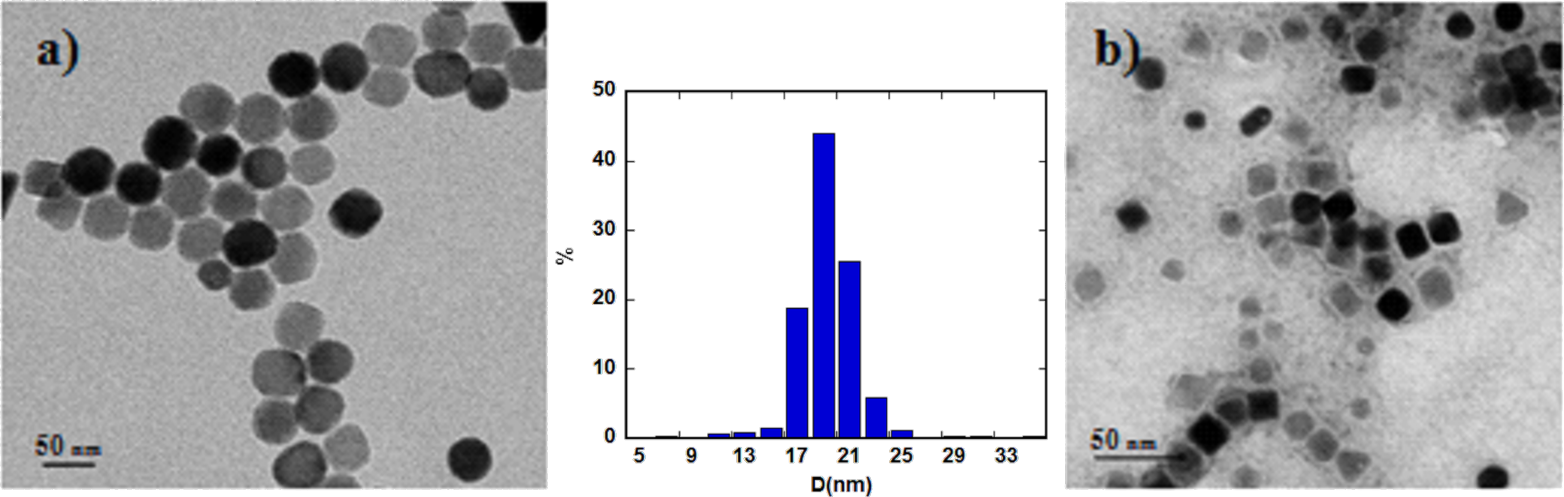
TEM images of a) Fe_3_O_4_@OA and b) Fe_3_O_4_@PMAO with the diameter size distribution shown between the images.

The TEM images of the nanoparticles corroborate this data and confirm that these nanoparticles are single crystals, discarding the appearance of twinning effects (Figure 1). The size distribution fits to a Gaussian profile with a mean size of 19 ± 2 nm for the magnetic nuclei, which do not undergo changes when coating with PMAO polymer (Figure 1b), maintaining monodispersity and size distribution. To better define the degree of agglomeration of the nanoparticles in solution, DLS measurements were performed at 24 h. The hydrodynamic diameter for Fe_3_O_4_@OA (in toluene) and Fe_3_O_4_@PMAO (in a 1:10 PBS/H_2_O solution) samples was 57 and 78 nm, respectively. These values do not agree with the concept that 19 nm diameter NPs are surrounded by one layer of oleic acid or PMAO [37], indicating rather the presence of some kind of aggregation that increases with the complexity of the recovering ligands. In the process of incorporation of the RGD peptides, the hydrodynamic size for Fe_3_O_4_@PMAO_RGD (in a 1:10 PBS/H_2_O solution) nanoparticles increased until the particles reached a diameter of 111 nm. The zeta potential value at 24 h confirms the high stability of the aqueous solutions of Fe_3_O_4_@PMAO (−41 mV), but the stability decreases until −15 mV when RGD is adhered to the NPs.

Infrared spectra of the magnetic nanoparticles have been performed in order to determine the type of ligands surrounding the magnetic nuclei (see Supporting Information File 1, Figure S2). In the case of oleic acid-coated Fe_3_O_4_@OA NPs, the –CH_2_ symmetric and asymmetric stretching vibrations at 2852 and 2920 cm^−1^, together with the absorption band at 1053 cm^−1^ from C–O single bond stretching, reveals adsorption of the oleyl group on the surface. After coating with PMAO, the stretching modes of C–O and C=O were observed at 1220 cm^−1^ and 1722 cm^−1^, respectively [38]. This last absorption is related to the carboxyl groups in PMAO, resulting from the opening of anhydride rings, confirming the recovery of the nanoparticles by the amphiphilic ligand. The percentage of organic coating has been calculated by TGA measurements performed in Ar atmosphere. A weight loss of 8% was observed between 200 °C and 500 °C, which may account for the mass evolution of oleic acid and/or other organic ligands on the sample surface. Above 700 °C, a weight loss around 10% is observed, which could be ascribed to decomposition of generated carbonates. It can be observed that the increasing weight loss for the PMAO covered sample (60%) compared with the initial oleic-coated NPs (18%), is in good accord with an effective recovery when using the PMAO amphiphilic ligand (see Supporting Information File 1, Figure S3).

### Magnetic properties

**Figure 2:**
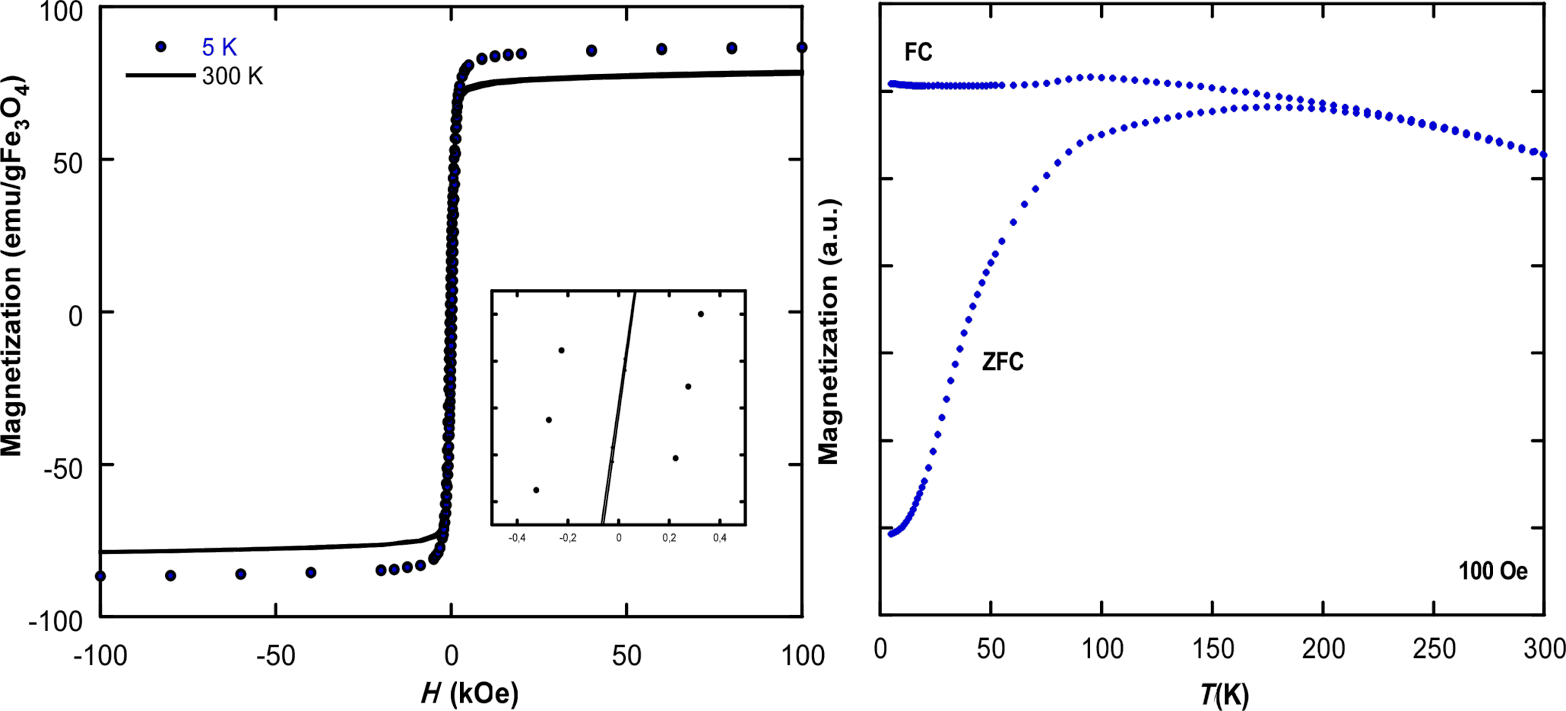
a) Experimental *M* vs *H* hysteresis cycles at 300 and 5 K and b) Magnetization of the zero-field-cooled (ZFC) and field-cooled (FC) Fe_3_O_4_@OA sample.

Magnetic hysteresis behavior was studied to estimate the magnetization of the Fe_3_O_4_@OA sample (Figure 2). The absence of a coercive field or remanence in the hysteresis loop recorded at 300 K is indicative of a superparamagnetic behavior of the sample. The saturation magnetization value obtained from the hysteresis loops at 300 K is 78.4 emu/g Fe_3_O_4_, which slightly deviates from the bulk saturation value of magnetite (92 emu/g) [39]. Nevertheless, the deviation could be ascribed to different effects such as crystallinity of the samples as well as the impact of surface spin disorder, which increases at high temperatures [40]. At 5 K, saturation magnetization increases (87.2 emu/g Fe_3_O_4_) to nearly reach the bulk value for magnetite. The coercive field value observed at 5 K is around 0.05 kOe, which is very similar to that observed in magnetite nanoparticles of similar size [41].

The measurement of magnetization versus temperature after cooling at zero-field-cooled (ZFC) and field-cooled (FC) for a colloidal sample dispersed in toluene is represented in Figure 2b. The evolution of the curves shows the usual characteristics of a superparamagnetic system, with a blocking temperature (*T*_B_) of 177 K and a progressive decrease of magnetization above *T*_B_. This high value of the blocking temperature is in good accord with the relatively large mean size of the NPs (around 19 nm) taking into account that *T*_B_ = *K*·*V* / 25·*k*_B_, where *K* is the effective magnetic anisotropy constant, *V* is the mean volume of the NPs, and *k*_B_ is the Boltzmann constant [42]. Nevertheless, the broad maximum observed in the ZFC branch is related to the contribution of nanoparticles of different sizes, as well as to the effects of interparticle interactions. Moreover, a sharp feature at 97 K due to the Verwey transition is observed. Although this Verwey transition occurs at 120 K in bulk magnetite, lower values are found in NPs, attributed to size effects and the crystallinity of the samples [43].

**Figure 3:**
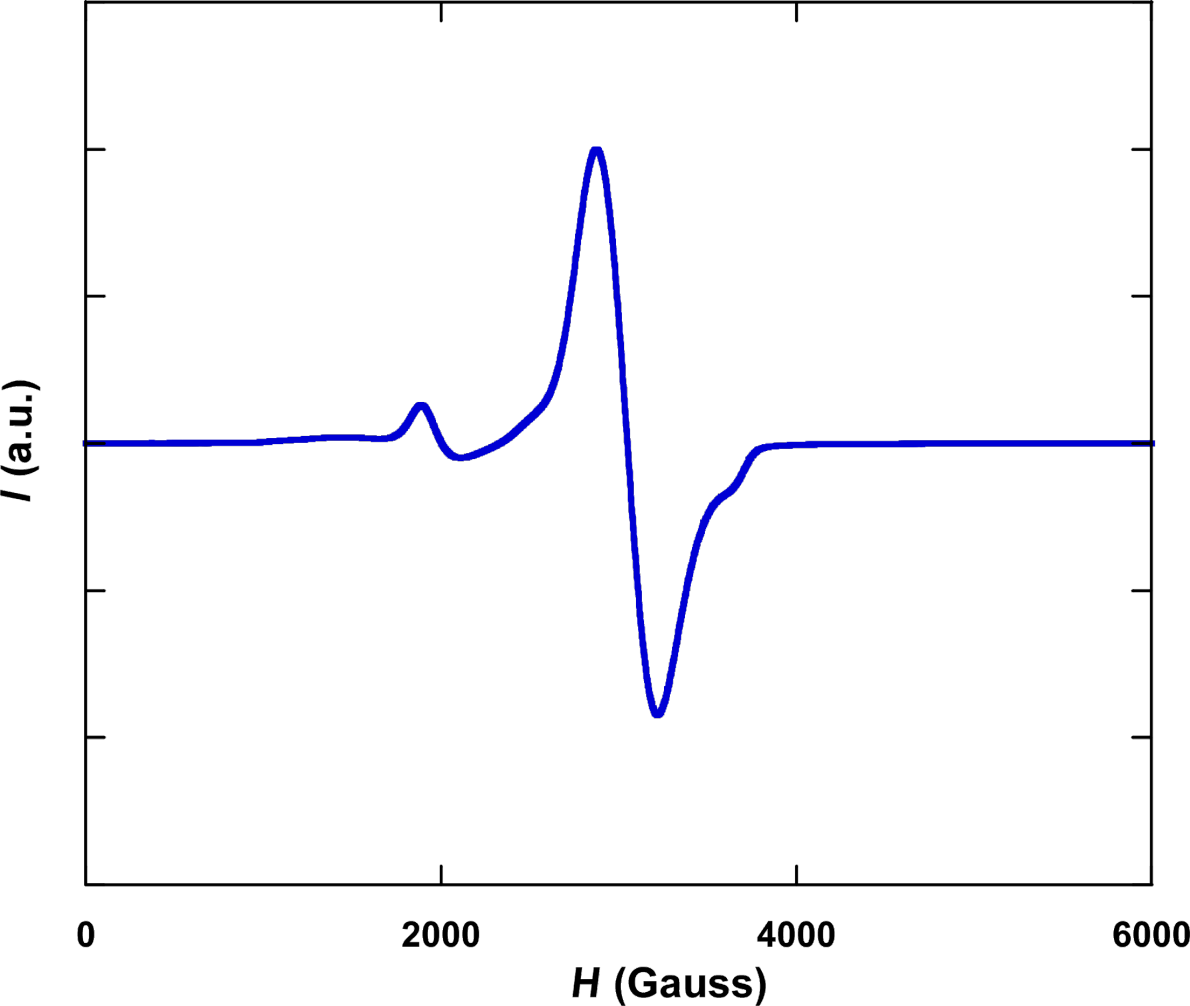
EMR spectrum at room temperature for the Fe_3_O_4_@OA sample measured in toluene.

More information concerning the magnetic characteristics of the samples can be obtained from the spectrum of electronic magnetic resonance (EMR) spectroscopy recorded at room temperature in colloidal medium (Figure 3). The spectrum exhibits the principal and a well-resolved line that fits to a Lorentzian profile with a *g*_eff_ value of 2.2. The *g*_eff_ value is higher than those observed in magnetite nanoparticles of smaller sizes and is in good agreement with the previously observed correlation between both parameters [44], confirming that the size of the nanoparticles can be easily estimated by this technique using a simple exponential function. Nevertheless, another small signal around *H* = 2000 G (3.5*g*) shows the existence of a different magnetic contribution explained by the coexistence of larger NPs, as can be noticed by the distribution observed in TEM photographs.

**Figure 4:**
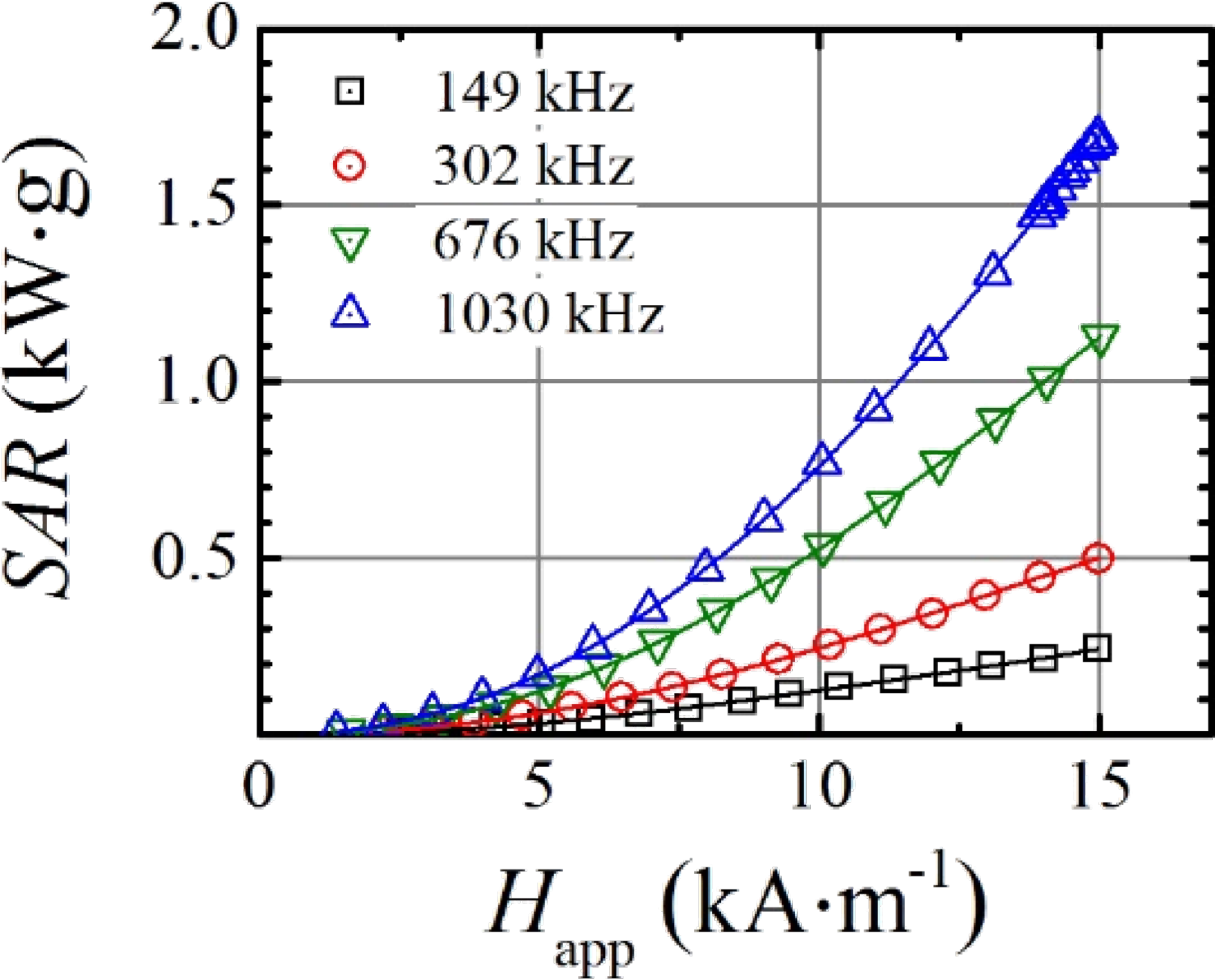
SAR values versus applied magnetic field intensity (*H*_app_) at different field frequencies for the Fe_3_O_4_@PMAO_RGD water dispersed nanoparticles.

As could be predicted from these results, high hyperthermia values should be expected from functionalized magnetic nanoparticles. SAR measurements were performed by the described AC magnetometer by varying the magnetic field amplitude (1–15 kA/m) at different frequencies (149, 302, 676 and 1030 kHz) for toluene dispersions of Fe_3_O_4_@OA and water dispersions of Fe_3_O_4_@PMAO and Fe_3_O_4_@PMAO_RGD. Above 600 kHz and 10 kA/m, all the dispersions present significant response, higher than 500 W/g. Taking into account that RGD-functionalized MNPs will be the only ones used for ulterior intra-arterial administration, the as measured SAR values for the Fe_3_O_4_@PMAO_RGD MNPs dispersed in water are represented in Figure 4. When performing the in vivo magnetic hyperthermia experiments, the magnetic field frequency was set to 606 kHz because at this frequency the NPs present a SAR high enough to create hyperthermia conditions. Precisely at 14 kA/m field intensity and 606 kHz field frequency, a linear interpolation gives a SAR value of 900 W/g for the measured sample.

### Intra-arterial administration of MNP suspensions and hyperthermia induction

The experimental studies to evaluate the capacity of the magnetic nanoparticles to induce hyperthermia were carried out in 16 rats bearing hepatic implants of colon adenocarcinoma. The development of the liver implants and their volumes were controlled and quantified by ultrasound. The suspensions of the Fe_3_O_4_@PMAO_RGD nanoparticles were administered through the splenic artery into the hepatic artery in a group of eight rats (MNpG), without observation of aggregation or arterial thrombosis in any of the animals. The other eight rats were intra-arterially infused with saline and used as controls (SG). The variables studied in the two groups of rats are shown in Table 1.

**Table 1:** Results of the measurements in the two groups of rats. SG: Control group, eight rats infused with saline. MNpG: group of eight rats infused with MNPs. ∆*T*ª: temperature increase quantified at the end of the hyperthermia induction cycle. Tumour necrosis (%): percentage of tumor necrosis established by pathologic analysis. The values are expressed as the mean (±SD) when they are adjusted to a Gaussian distribution or median (range) otherwise.

		SG (*n* = 8)	MNpG (*n* = 8)	*p*-value

Weight (g)		269 (±18.9)	252 (±20.2)	0.11
Tumor volume (mm^3^)		0.41 (±0.22)	0.46 (±0.19)	0.42
*T*ª tumor (°C)				
	∆*T*ª Highest	4.66 (±0.99) 41 (32.9–42.9)	7.89 (±1.2) 43 (40.0–43.7)	<0.05
*T*ª rectum (°C)				
	∆*T*ª Highest	2.45 (±0.82) 34.9 (33.7–37.0)	2.39 (±1.4) 34.5 (32.0–36.8)	0.73
*T*ª liver (°C)				
	∆*T*ª Highest	4.73 (±0.98) 41.4 (38.1–42.6)	7.76 (±0.63) 43 (39–43)	<0.05
ICP–MS [Fe] (µg·g^−1^)				
	Tumor Liver	51 (±7.38) 198 (±15.5)	93 (±35.56) 342 (±79.0)	<0.05 <0.05
Tumor necrosis (%)		4 (1–13)	20 (3–99)	<0.05

Regarding the proportion of the animal weight and tumor volume of the hepatic implants, the differences between the two groups were not statistically significant.

Hyperthermia was induced in the rats by applying a magnetic field intensity of 14 kA/m until a liver temperature of 43 °C was reached and then this temperature was maintained for 21 min. In all the animals exposed to the electromagnetic applicator (EA), the body temperature remained within safe margins, and rapidly reverted to normal values after returning the animal to its cage. To evaluate the thermal induction, three probes (rectum, tumor and liver of each rat) monitored temperature changes during the induction cycles. The application of the alternating magnetic field increased the body temperature (rectum) around two degrees, with no significant difference between SG and MNpG animals. The thermal increase registered in tumors of SG rats was of 4.66 ± 0.99 °C, while in MNpG animals it was of 7.82 ± 1.2 °C, the difference being statistically significant (*p* < 0.05). The liver temperature showed values quite similar to those observed in tumors, both in SG animals (thermal increase 4.73 ± 0.98 °C) and in MNpG rats (thermal increase 7.76 ± 0.63 °C), the difference being statistically significant (*p* < 0.05). Therefore, the temperature of the liver and the tumor increased around 3 °C more in MNpG-infused animals than in SG ones. This difference should be attributed to the presence of exogenous iron in those tissues. The fact that in SG rats (without MNPs) an increase of more than 4.5 °C was observed both in the liver and the tumor could be explained by the eddy currents induced by the EA. On the other hand, the fact that the body temperature of all the animals was only raised by 2.4 °C could be due to the dispersion of heat by the blood flow through those parts of the animal not within the focus of the EA.

After hyperthermia, the rats were sacrificed and tumor and liver tissue samples were obtained for quantification of iron content and for pathologic analyses. The iron concentration [Fe] in micrograms per gram (µg·g^−1^) was measured in samples of hepatic and tumor tissue by inductively coupled plasma mass spectrometry (ICP-MS). The infusion of MNPs significantly increased [Fe] in the liver of MNpG rats as compared to quantifications obtained in SG animals (342 µg·g^−1^ vs 198 µg·g^−1^; *p* < 0.05). If we focus on tumors, a similar behavior may be observed, but with quite lower values (93 µg·g^−1^ vs 51 µg·g^−1^; *p* < 0.05). The data of [Fe] quantified in SG rats were similar to results obtained in previous experiments performed by our group, from rats bearing tumor implants of colorectal carcinoma and not infused with MNPs [1]. Following MNP infusion, the iron content increased by 1.7-fold both in tumor and liver tissue. This fact could be related to the presence of the exogenous iron from the magnetic nanoparticles. Furthermore, this exogenous iron could have been responsible of the greater thermal increase detected in MNpG animals.

**Figure 5:**
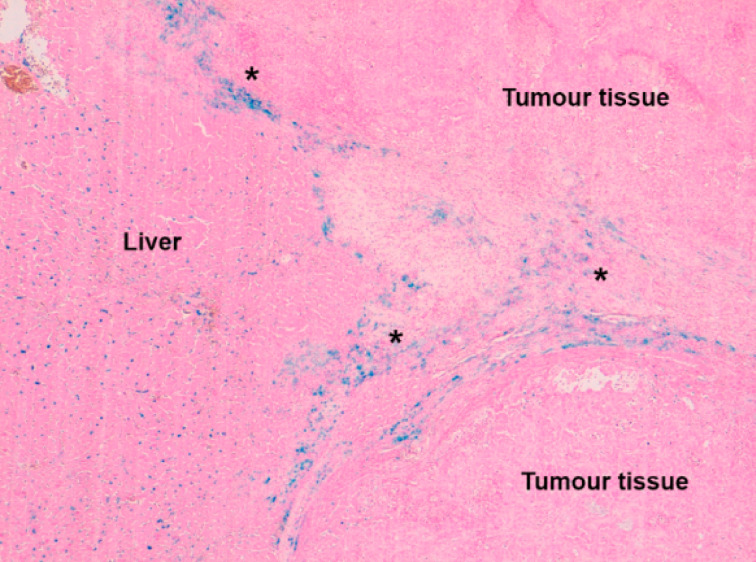
Kupffer cells with phagocytized iron deposits (blue dots) in liver tissue. In the fibro-vascular tissue matrix around the tumor (asterisks) blue iron deposits are observed.

In order to know the tumor damage induced by hyperthermia, a skilled pathologist carried out the morphological assessment of the samples, performing a visual qualitative analysis of tissue necrosis. Pathologic studies with Perls’ Prussian Blue stain concluded that deposits of iron could be observed within hepatic tissue, as blue dots corresponding to iron phagocytosis by Kupffer cells. On the other hand, when observing the tumor tissue, iron was seen as scattered deposits within the peripheral fibro-vascular matrix (Figure 5).

This fact could be explained by the special affinity of the RGD peptides adhered to the surface of our MNPs, for integrin α_V_β_3_ [45–46]. It has been established that α_V_β_3_, α_V_β_5_ and α_V_β_1_ integrins are membrane cell receptors related to angiogenesis and intercellular adhesion, and are overexpressed in some types of solid tumors [47]. So it could be suggested that α_V_β_3_ integrin would be responsible for the special location of MNPs around the tumors.

**Figure 6:**
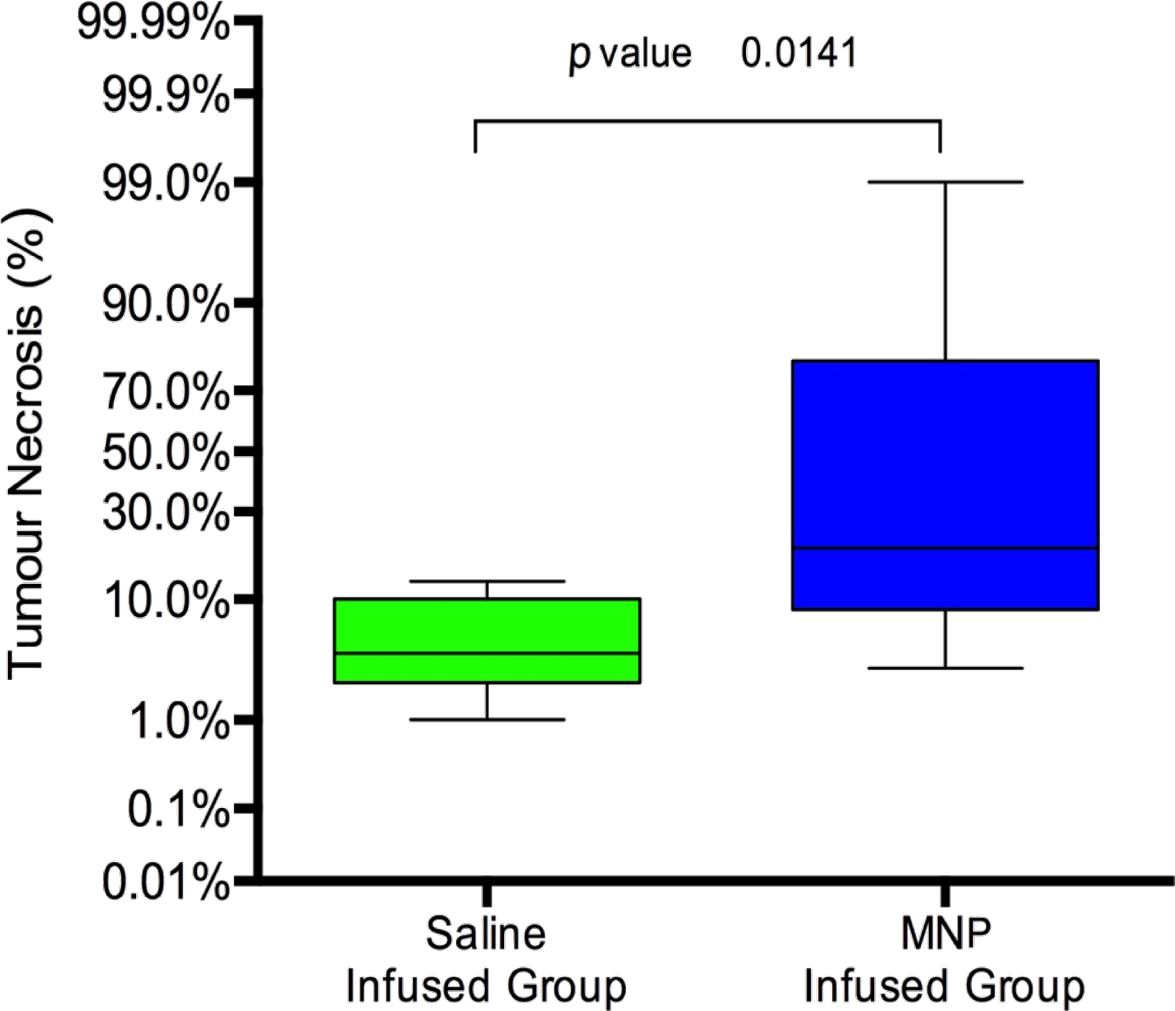
Error bars show the percentages of tumor necrosis in rats infused with saline or magnetic nanoparticles. A significant difference can be observed between the two groups of rats (*p* = 0.01).

Pathological studies demonstrated that there was no relevant damage in hepatic tissue after exposure to the hyperthermia treatment. However, tumor necrosis was observed, especially in MNpG rats, in spite of temperature increases being very similar in liver and tumor tissues. SG animals showed a median value of tumor necrosis of 4% (range: 1–13%). These minimal necrosis changes must be considered as natural degeneration phenomena in relation with tumor growth. Nevertheless, in MNpG animals, the mean percentage of necrotic tumor measured by pathologic analyses was increased by 5-fold, reaching 20%; however, these rats showed a very wide range of tissue necrosis (3–99%) (Figure 6).

The fact that nearly no damage could be observed in normal liver tissue could be due to different phenomena. First, the portal blood flow could effectively behave as a heat interchanger, cooling the tissue heated by the MNPs. Moreover, magnetite nanoparticles once internalized into the cell would be quickly modified by the lysosomes and lose their capacity to be moved by the HVMC, which dramatically would reduce their heating capability [48–49]. On the other hand, the necrosis produced in tumors of MNpG rats could be ascribed to several mechanisms working together. Even though tumor cells are more sensitive to hyperthermia than healthy ones, the temperature increase verified in MNpG animals was not high enough to induce the homogeneous damage in all the tumors. In pathological samples, scattered deposits of iron within the fibro-vascular matrix of the tumor periphery were observed. These deposits could be the key to achieve suitable and homogeneous tumor necrosis. We believe that in rats with enough amounts of MNPs deposited in the matrix, it is possible to achieve tumor necrosis; but in rats with similar temperature increase quantified into tumors, but less amount of MNPs fixed to the matrix, it is not possible to induce suitable damage to the tumors. Therefore, the achievement of an effective tumor necrosis would require increasing the amount and affinity of the MNPs towards the peripheral fibro-vascular matrix.

## Conclusion

The synthesis method of thermal decomposition by successive additions produced samples of very high crystallinity without impurities with an average particle diameter of 19 nm. This was corroborated by TEM and DLS, indicating that the RGD-functionalized MNPs reach a hydrodynamic size large enough to remain in the bloodstream to reach the tumor [50]. The high-saturation magnetization achieved by the NPs precludes high hyperthermia values. SAR values above 900 W/g were obtained for the Fe_3_O_4_@PMAO_RGD MNPs dispersed in water at 14 kA/m and 606 kHz, where these NPs were selected as heating agents for in situ magnetic fluid hyperthermia protocols.

The surgical model employed has proved useful to selectively deliver different fluids containing Fe_3_O_4_@PMAO_RGD MNPs to the liver with colorectal tumor. A histological analysis showed that MNPs were distributed through the arterial vessels of the liver, without conforming aggregates or thrombosis. Nevertheless, the observation of MNPs as blue tags corresponding to Kupffer cells indicates that MNPs could not avoid phagocytosis. The fact that MNPs could be found in the fibro-vascular tissue surrounding the tumors could be explained by the special affinity of the RGD peptides adhered to the surface of our MNPs for integrin α_V_β_3_.

The exposure of the animals to hyperthermia treatment yielded an incremental temperature of around 3 °C more in animals infused with MNPs, than in control animals, both in liver tissue and in tumor tissue. This difference should be attributed to the presence on exogenous iron in those tissues. Nevertheless, there was no relevant damage in hepatic tissue, only in tumorous tissue; and while in some animals nearly all the tumor was destroyed, others showed very low damage in tumor tissue. In this way, the necrosis observed in the tumors could be due to damage caused to the peritumoral vascular bed by hyperthermia. However, differences in tumor size, energy applied, or duration of the exposure could not be correlated with percentage of tumor necrosis.

Further experiments would be needed to clarify the mechanism through which necrosis is achieved in order to improve the therapy. However, we find our results quite promising as the first steps to develop a new therapeutic approach for poorly vascularized liver tumors/metastases based on arterially delivered MNPs and EA-induced local hyperthermia have been accomplished.

## Experimental

### Fe_3_O_4_ NP synthesis

The nanoparticles were synthesized by optimization of a previously reported method based on successive additions of iron pentacarbonyl and posterior decomposition in benzyl ether [44,51]. A mixture of Fe(CO)_5_ (3 mmol), 1,2-hexadecanediol (5 mmol), oleic acid (4 mmol), oleylamine (6 mmol) and benzyl ether (25 mL) were added to a three-neck flask. Then, the reaction mixture was heated under mechanical stirring and a flow of argon gas until a temperature of 140 °C was reached. This temperature was kept for 30 min and then the solution was heated to reflux (280 °C) for 120 min under argon. The second, third and fourth additions were made by adding a mixture of Fe(CO)_5_ (9, 10 and 15 mmol), oleic acid (5, 8 and 9 mmol) and oleylamine (6, 6 and 6 mmol) to the flask, respectively [52–53]. Subsequently, the solutions were cooled to room temperature, cleaned by magnetic decantation and resuspended in toluene to obtain a colloidal dispersion of NPs named Fe_3_O_4_@OA.

### Synthesis of Fe_3_O_4_@PMAO_RGD NPs

The particle surface was modified with poly(maleic anhydride-alt-1-octadecene (PMAO) [54–55]. The phase transfer was made by mixing 20 mg Fe_3_O_4_ nanocrystals and 400 mg PMAO in 200 mL chloroform and sonicated at room temperature (1 h). After removing the chloroform, the nanoparticles were resuspended and shaken in 20 mL of NaOH 0.1 M and 20 mL distilled H_2_O at 60 °C. The polymer surface was hydrolized and free carboxylic groups were obtained [56]. Finally, these hydrosoluble nanoparticles, named Fe_3_O_4_@PMAO, were passed through a 0.2 μm filter. Carboxylic groups of PMAO were modified with an amino modified linker (10-(2-cyclooctyn-1-oxy)-3-aza-5,8-dioxa-4-oxodecyl-1-amine) (cyOct) to add an alkyne group [57–60]. First, 8 mg of MNPs were added in a total of 0.5 mL SSB (H_3_BO_3_/ Na_2_B_4_O_7_·10H_2_O) 50 mM pH 9. Then, 0.01 mg of *N*-(3-dimethylaminopropyl)-*N*′-ethylcarbodiimide hydrochloride (EDC) and 0.001 mg of 2-(2-cyclooct-2-yn-1-yloxy)ethoxyethyl(2-aminoethyl) carbamate were added to the mixture in order to provide a new functionality and 0.01 mg of tris(hydroxymethyl)aminomethane (TRIS) to block the superficial charges. Finally, the alkyne modified NPs were coupled by the molecule of interest, the azide-modified RGD peptide (Arg-Gly-Asp) (RGD-N_3_) in water in a one-step procedure by a click reaction (see Supporting Information File 1). These “clicked” MNPs (Fe_3_O_4_@PMAO_RGD) were thoroughly washed with distilled water and MNP fluids with concentrations around 1–1.5 mg Fe/mL in a PBS/H_2_O (1:10) buffer were prepared to perform in vivo assays.

### NP characterization

**Figure 7:**
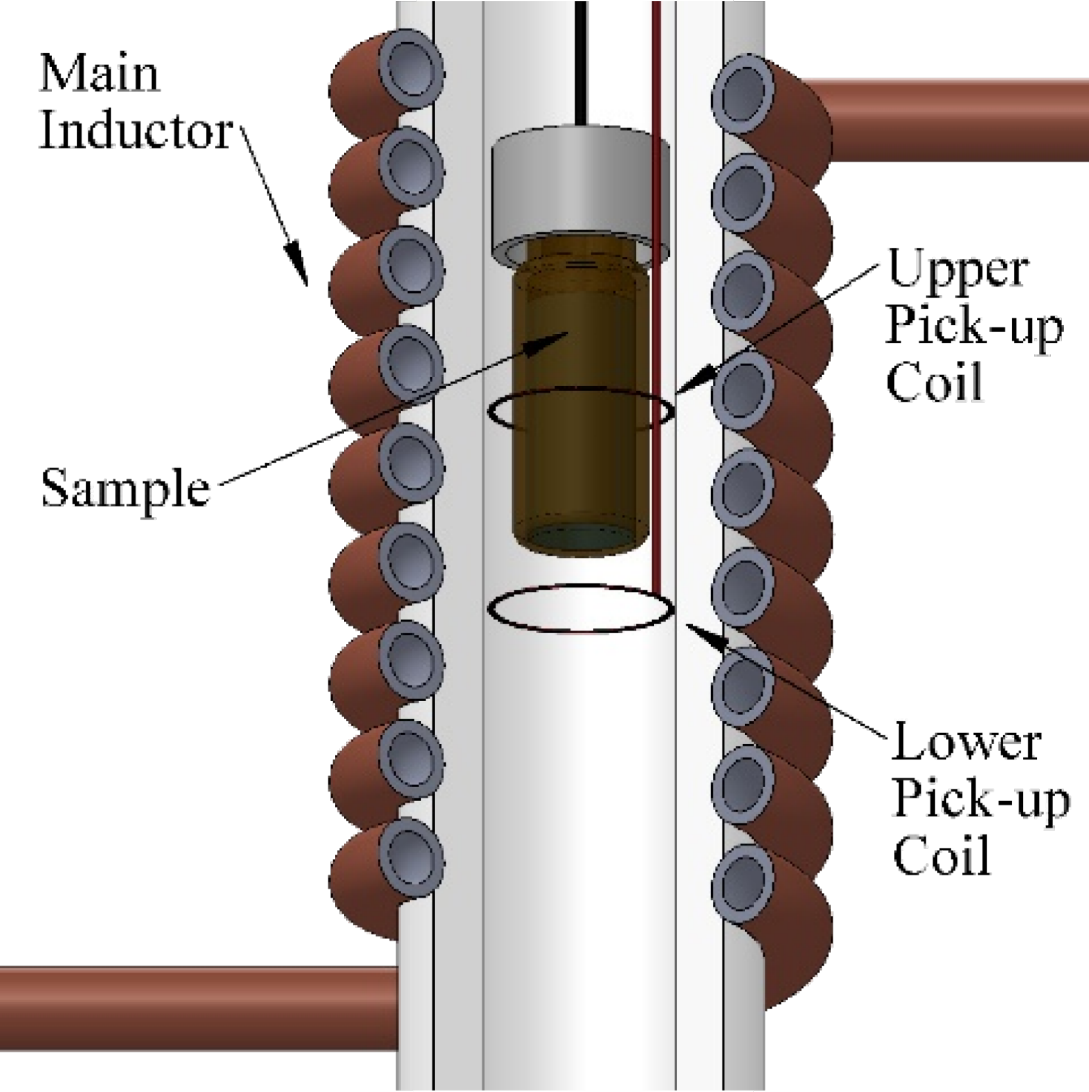
Scheme of the AC magnetometer with the air-core coil and the oppositely wound pick-up coils.

X-ray diffraction (XRD) of powder samples was obtained using a Philips PW1710 diffractometer equipped with copper anodes. The X-ray source operated at 40 kV and 40 mA and the 2θ scan was performed in the 10° < 2θ < 90° range every 0.02° with a scan step speed of 1.25 s. Thermogravimetric measurements were performed in a NETZSCH STA 449 C thermogravimetric analyzer by heating ≈10 mg of sample at 10 °C/min under dry Ar atmosphere. The particle size and morphology was determined from TEM micrographs in a Philips CM200 microscope at an acceleration voltage of 200 kV. Dynamic light scattering (DLS) measurements were carried out at 25 °C with a Nano ZS (Malvern Instruments) equipped with a solid-state He-Ne laser (λ = 633 nm) to determine the hydrodynamic diameter of the hydrosoluble NPs. The FTIR spectra of the nanoparticles and ligands were collected on a FTIR-8400S Shimadzu spectrometer in the range of 4000–400 cm^−1^. The measurements of magnetization versus temperature at 10 Oe were carried out at 5 and 300 K using a Quantum Design MPMS-7 SQUID magnetometer. The hysteresis loops at room temperature were collected in a homemade VSM magnetometer up to a maximum field of 18 kOe with high low field resolution. The hysteresis loops at 5 K were performed in a VSM magnetometer from Cryogenic Ltd. up to a maximum field of 100 kOe. Additionally, the SAR values of the synthetized NPs were measured by a lab-made AC magnetometer previously described in the literature [61]. The necessary AC magnetic field was generated by a water-cooled air-core inductor, which is a part of a resonant LCC circuit fed by a linear power amplifier. The AC magnetometer is made of two pick-up coils wound in opposite directions (Figure 7). The SAR values were measured at different AC magnetic field intensities (0–15 kA∙m^−1^) and frequencies (149, 303, 676 and 1030 kHz).

The hyperthermia treatments for NP-injected animals were performed inside a second lab-made EA especially designed and constructed for experiments with small laboratory animals (see Supporting Information File 1, Figure S4). The frequency was fixed at 606 kHz until 43 °C was registered in the liver probe; thereafter, the intensity was automatically adapted by an on–off controller in order to maintain the liver temperature at 43 °C until 21 min of treatment were completed.

### Development of the experimental model

The study was carried out on three-month-old WAG/RijCrl male rats. The rats were kept in an animal house under standard conditions. All the procedures were performed in accordance with current national legislation on animal experimentation and approved by the ethics committee of our Institution. To induce liver colorectal tumors, under 1.5% isoflurane anaesthesia, 25,000 syngeneic cells of colon adenocarcinoma (cellular line: CC-531) suspended in 0.05 mL of Hank’s solution were inoculated into the left hepatic lobe through a subcapsular injection [1]. On day 35, the rats were studied by ultrasonography to evaluate tumor development. Ultrasonograms were performed using an 18 MHz probe and were carried out under isoflurane anesthesia. The tumor volume of each rat was quantified from echographic measurements of the three largest diameters, axial, coronal and sagittal. Animals not bearing tumors were discharged. The study was developed on 16 rats that were randomly allocated into two groups:

- Saline group (SG): Eight rats to be infused intra-arterially with 1 mL of saline, to assess the effects of the infusion procedure on tumor implants.
- MNp group (MNpG): Eight rats to be infused intra-arterially with the prepared nanoparticles.

### Liver infusion procedure

Anesthesia was induced with diazepam (15 mg/kg i.p.), ketamine (80 mg/kg i.p.) and medetomidine (0.5 mg/kg i.p.). Through a midline laparotomy, the celiac trunk and the splenic artery were identified and dissected. Using a surgical microscope (Leika M651), a microcannula (0.014 in OD) was inserted through the splenic artery into the hepatic artery, and a Yasargil clip was placed to stabilize it. Using an infusion pump, 1 mL of saline or magnetic NP fluid (PBS with Fe_3_O_4_@PMAO_RGD, to be administered at a dose of 5 mg Fe/kg) was infused (0.15 mL/min). Once the microcannula was removed, a haemostatic gelatin sponge was firmly applied on the puncture site for five minutes. Without removing the haemostatic agent, the laparotomy was closed, and meloxicam (2 mg/kg sc) plus buprenorphine (0.05 mg/kg sc) were administered.

### Heat induction procedure

Eighteen hours later, under diazepam/ketamine/medetomidine anaesthesia, three thermic probes were inserted (inside the tumor, between liver lobes and inside the rectum). Immediately after, the animals were placed inside a second lab-made EA especially designed and constructed for experiments with small laboratory animals (see Supporting Information File 1).

### Necropsy and pathological studies

Within the first 24 h following hyperthermic treatment, while under isofluorane anaesthesia, the liver was removed and samples of hepatic and tumor tissues were obtained. The iron content, [Fe], in μg/g of wet tissue was assessed in frozen samples by ICP–MS. From samples embedded in paraffin, sections were obtained and stained with hematoxylin-eosin and Perls’ Prussian Blue. During tumor growth, areas of tissue necrosis appear within the implants, and their size directly correlates with tumor volume. These necrosis foci are undistinguishable from those induced by hyperthermia or any other treatment. A skilled pathologist assessed the percentage of hepatic or neoplastic necrosis by visual qualitative analysis of the blinded samples. The same pathologist evaluated the presence of iron deposits stained with Prussian Blue.

### Statistical analysis

Quantitative variables are described by their mean and standard deviation (SD) or by their median and range. Statistical analysis was carried out using nonparametric and nonpaired tests: Mann Whitney U test. For two-variable data sets, we produced box plots where *p*-values < 0.05 were considered significant.

## Supporting Information

File 1 Additional experimental information.
The Supporting Information includes a representative scheme of the functionalization method of Fe_3_O_4_ NPs, a description of the preparation of RGD peptide, the heat induction procedure and additional figures of the XRD, TGA and FTIR characterization.
