# Supplementary material for: Antitumor magnetic hyperthermia induced by RGD-functionalized Fe3O4 nanoparticles, in an experimental model of colorectal liver metastases
Source: Beilstein J Nanotechnol. 2016 Oct 28;7:1532–42. doi: 10.3762/bjnano.7.147 (PMC5238624; doi:10.3762/bjnano.7.147)
Supplement: File 1 — Additional experimental information. The Supporting Information includes a representative scheme of the functionalization method of Fe3O4 NPs, a description of the preparation of RGD peptide, the heat induction procedure and additional figures of the XRD, TGA and FTIR characterization. [file Beilstein_J_Nanotechnol-07-1532-s001.pdf]

Supporting Information

for

Antitumor magnetic hyperthermia induced by RGD-functionalized Fe_3O_4 nanoparticles, in an experimental model of colorectal liver metastases

Oihane K. Arriortua[1], Eneko Garaio[1], Borja Herrero de la Parte[2], Maite Insausti[*1,3], Luis Lezama[1,3], Fernando Plazaola[1], Jose Angel García[1,3], Jesús M. Aizpurua[4], Maialen Sagartzazu[4], Mireia Irazola[1], Nestor Etxebarria[1], Ignacio García-Alonso[2], Alberto Saiz-López[5], and José Javier Echevarria-Uraga[5]

Address: [1]Faculty of Science and Technology, University of the Basque Country, UPV/EHU, 48080, Bilbao, Spain, [2]Faculty of Medicine and Nursing, University of the Basque Country, UPV/EHU, P.O. Box 644, 48080, Bilbao, Spain, [3]BCMaterials, Basque Center for Materials, Applications and Nanostructures, 48160, Derio, Spain, [4]José Mari Korta Center, University of the Basque Country, UPV/EHU, 20018 Donostia, Spain, and [5]Galdakao Usansolo Hospital, 48960 Bizkaia, Spain

Email: Maite Insausti - maite.insausti@ehu.eus

* Corresponding author

Additional experimental information

This file includes:

Scheme S1. Representative scheme of the functionalization method.

Figures S1–S4. Additional figures of DRX, TG, IR and magnetic field intensity inside the inductor.

1. Synthesis of the azide modified RGD, H-Arg-Gly-Asp-NH-$(CH_2CH_2O)_3CH_2CH_2N_3$ (RGD-N_3).
2. Description of the heat induction procedure.

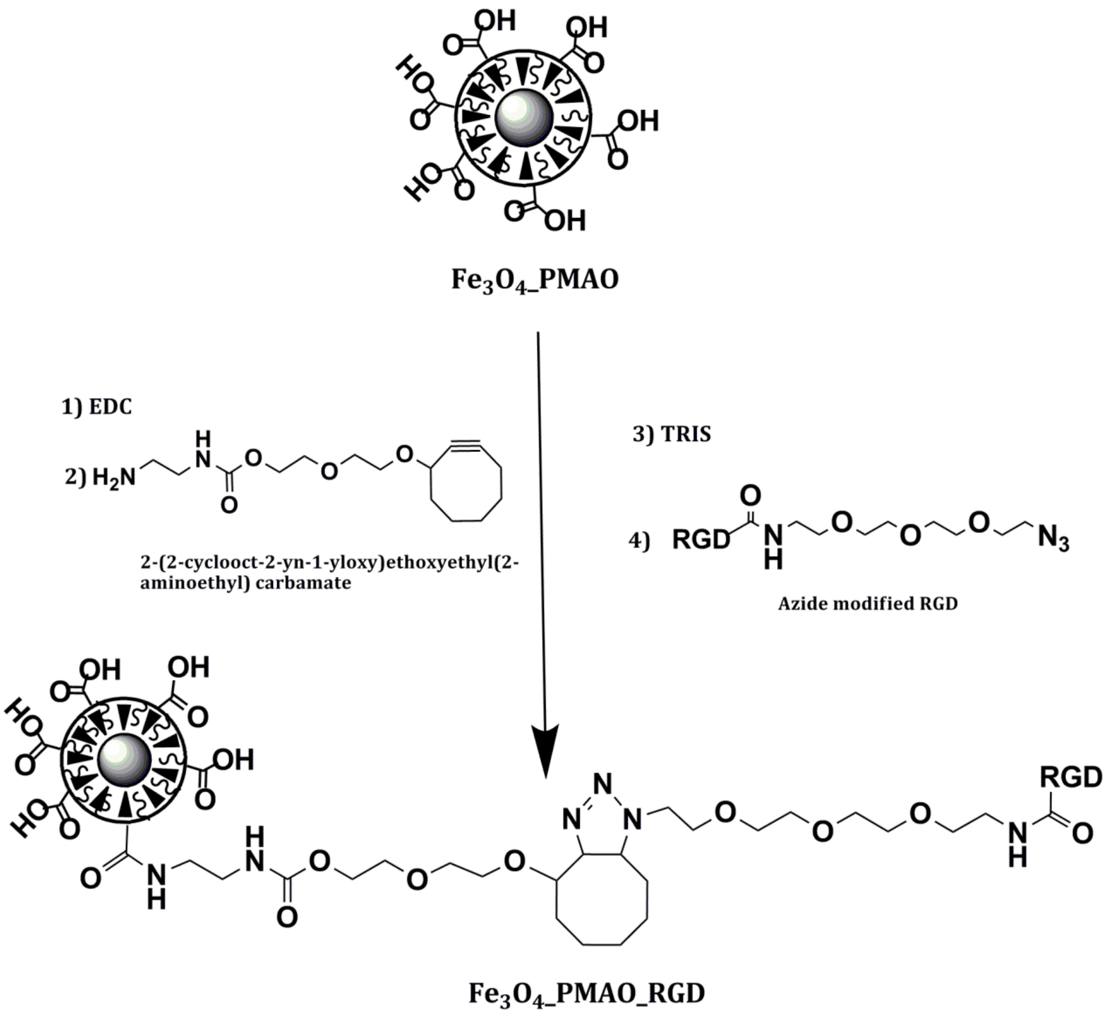

Scheme S1: Schematic functionalization of Fe_3O_4 NP surface.

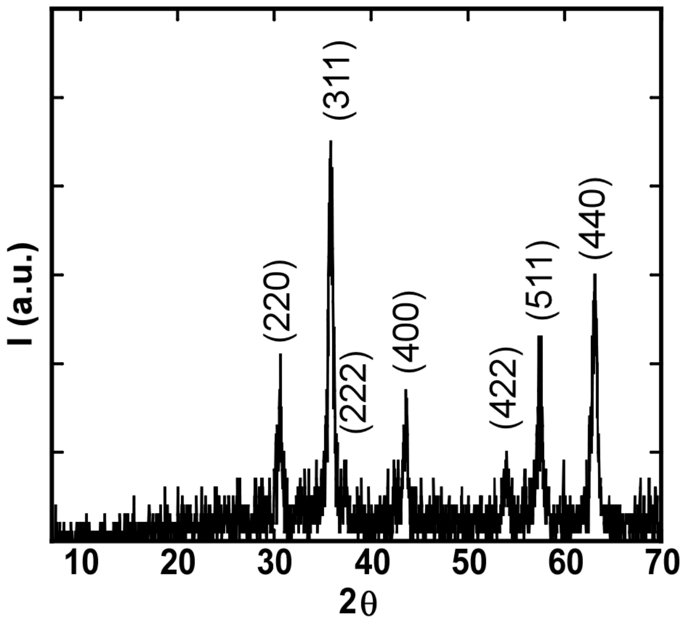

Figure S1: X-ray diffraction pattern of Fe_3O_4 NPs.

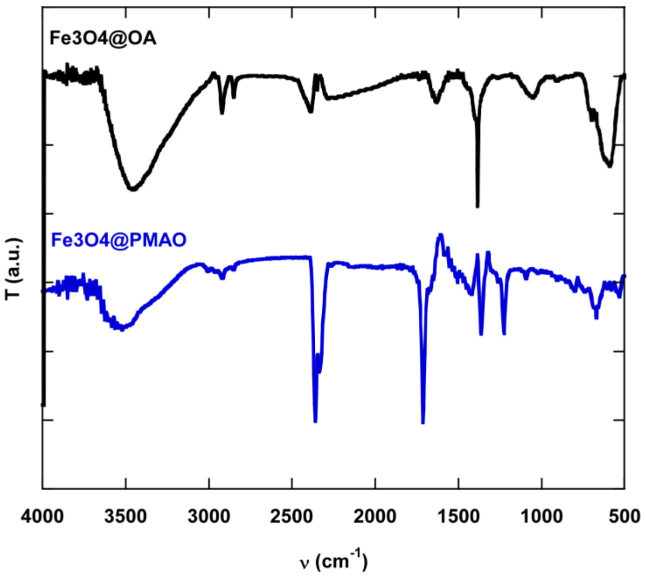

Figure S2: FTIR spectrum of Fe_3O_4@OA and Fe_3O_4@PMAO NPs.

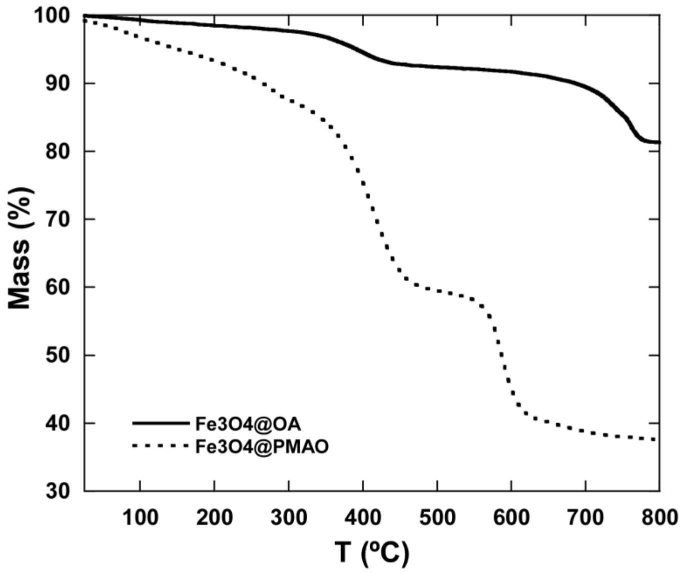

Figure S3: Thermogram of Fe_3O_4@OA and Fe_3O_4@PMAO NPs.

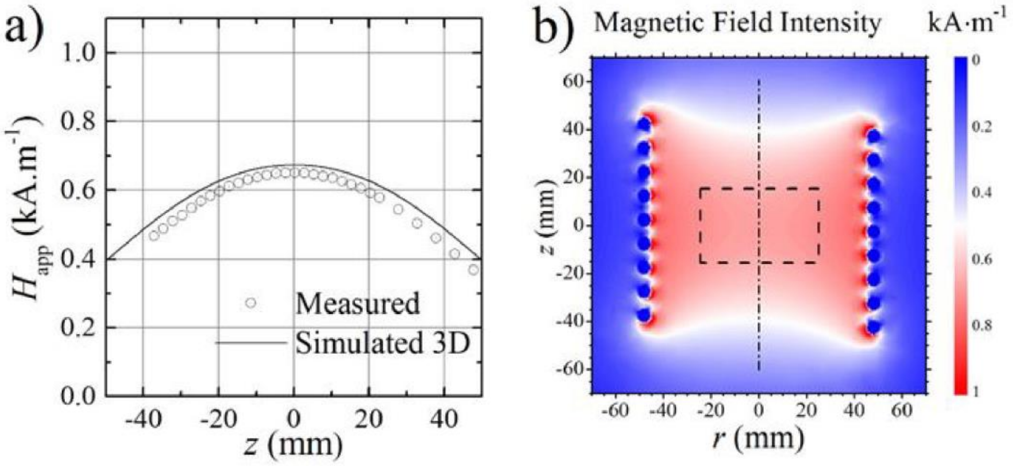

Figure S4: a) Z component of magnetic field intensity through coil axis simulated by FEM (solid line) and measured by a scout coil (empty circles). b) Magnetic field intensity distribution calculated by 3D FEM. Dashed box indicates the cylinder where the field is homogeneous. An AC electrical current of 10 A intensity flows across the inductor.

1. Synthesis of the azide modified RGD, H-Arg-Gly-Asp-NH-$(CH_2CH_2O)_3CH_2CH_2N_3$ (RGD-N_3)

A solution of Boc-Arg(Pbf)-GlyOH (0.34 mmol, 200 mg) and H-Asp(O*t*-Bu)-NH-$(CH_2CH_2O)_3CH_2CH_2N_3$ (0.34 mmol, 133 mg) in anhydrous CH_2Cl_2 (12.3 mL) was cooled to 0 ºC. Netx, EDC·HCl (0.51 mmol, 98 mg), HOBT·H_2O (0.51 mmol, 69 mg) and triethylamine (0.51 mmol, 92 µL) were added and the mixture was stirred at room temperature for 16 h. The solvent was evaporated at reduced pressure and the resulting crude Boc-Arg(Pbf)-Gly-Asp(O*t*-Bu)-NH-$(CH_2CH_2O)_3CH_2CH_2N_3$ product was purified by column chromatography (silicagel, CH_2Cl_2/MeOH 95:5). Yield: 214 mg (65%). Compound Boc-Arg(Pbf)-Gly-Asp(O*t*-Bu)-NH-$(CH_2CH_2O)_3CH_2CH_2N_3$ (0.05 mmol, 50 mg) was dissolved in trifluoroacetic acid (0.16 mmol, 0.80 mL) and the mixture was stirred at 35 ºC for 3 h. The solvent was evaporated at reduced pressure and the resulting crude product was purified by precipitation in diisopropyl ether.

2. Description of the heat induction procedure

Eighteen hours later, under Diazepam-Ketamine-Medetomidine anaesthesia, three thermic probes were inserted (inside the tumour, between liver lobes and inside the rectum). These probes consist of commercial (OpSens) optical fibre thermometers, immune to radio-frequency magnetic fields. Immediately after, the animals were placed inside a second lab-made EA especially designed and constructed for experiment with small laboratory animals, which is based on a resonant LCC circuit fed by a power amplifier. The AC magnetic fields are generated by an air-core inductor. In order to ensure electrical stability and avoid resistive heating, the inductor was refrigerated by water flowing across the pipes, which were connected to an external refrigerator. The magnetic field intensity generated by this coil was calculated by finite element method (FEM) and measured by a mobile pick-up coil.

Figure S4a shows the so measured and calculated magnetic field intensities through the coil axis. According to the performed FEM calculations, in the centre of the coil there is a 3.1 cm length and 4.6 cm diameter cylinder where the field is at least 4% homogenous (Figure S4b). In all the experiments, the rats were placed in such a way that the liver was located in the centre of the coil, where the field is homogeneous.

Regarding to the applied AC magnetic fields during the performed hyperthermia experiments, the frequency was fixed at 606 kHz until 43 ºC were registered in the liver probe; thereafter, the intensity was automatically adapted by an on-off controller in order to maintain the liver temperature at 43 ºC until 21 min of treatment were completed. This controller consists on setting the field intensity to 14 kA/m if the liver temperature was below 43 ºC and, on the contrary, setting it to 6 kA/m if the temperature was above 43 ºC. Using all three probes, we obtained results regarding the

temperature increases achieved at the end of induction cycles, and the highest temperatures reached in the three different areas, rectum, liver and tumour.
